# Targeting the NCOA3-SP1-TERT axis for tumor growth in hepatocellular carcinoma

**DOI:** 10.1038/s41419-020-03218-x

**Published:** 2020-11-25

**Authors:** Wenbin Li, Yue Yan, Zongheng Zheng, Qiaohua Zhu, Qian Long, Silei Sui, Meihua Luo, Miao Chen, Yizhuo Li, Yijun Hua, Wuguo Deng, Renchun Lai, Liren Li

**Affiliations:** 1 Sun Yat-sen University Cancer Center; State Key Laboratory of Oncology in South China; Collaborative Innovation Center of Cancer Medicine, Guangzhou, China; 2 Institute of Neuroscience and Department of Neurology of the Second Affiliated Hospital of Guangzhou Medical University, Key Laboratory of Neurogenetics and Channelopathies of Guangdong Province and the Ministry of Education of China, Guangzhou, China; 3 The Third Affiliated Hospital, Sun Yat-Sen University, Guangzhou, China; 4 Shunde Hospital of Southern Medical University, Foshan, Guangdong, China; 5 Institute of Cancer Stem Cell, Dalian Medical University, Dalian, China

**Keywords:** Oncogenes, Tumour biomarkers

## Abstract

Hepatocellular carcinoma (HCC) has a high mortality rate and lacks an effective therapeutic target. Elevated expression of human telomerase reverse transcriptase (TERT) is an important hallmark in cancers, but the mechanism by which TERT is activated differentially in cancers is poorly understood. Here, we have identified nuclear receptor coactivator-3 (NCOA3) as a new modulator of TERT expression and tumor growth in HCC. NACO3 specifically binds to the TERT promoter at the -234 to -144 region and transcriptionally activates TERT expression. NCOA3 promotes HCC cell growth and tumor progression in vitro and in vivo through upregulating the TERT signaling. Knockdown of NACO3 suppresses HCC cell viability and colony formation, whereas TERT overexpression rescues this suppression. NCOA3 interacts with and recruits SP1 binding on the TERT promoter. Knockdown of NCOA3 also inhibits the expression of the Wnt signaling-related genes but has no effect on the Notch signaling-targeting genes. Moreover, NCOA3 is positively correlated with TERT expression in HCC tumor tissues, and high expression of both NCOA3 and TERT predicts a poor prognosis in HCC patients. Our findings indicate that targeting the NCOA3-SP1-TERT signaling axis may benefit HCC patients.

## Introduction

Hepatocellular carcinoma (HCC) is one of the most common cancers and a leading cause of cancer death worldwide^1^. The rising incidence and mortality rate of HCC in most countries are due to an increased rate of chronic Hepatitis C virus infection, alcohol abuse, and obesity^2,3^. Although therapeutic approaches for HCC have been improved in recent years, the long-term prognosis of patients with HCC is still poor^4^. The molecular mechanisms leading to the development of HCC are complicated, consist of various genetic and epigenetic changes, and are associated with altered signaling pathways^5^. Among these molecular events, elevated expression of human telomerase reverse transcriptase (TERT) is an important alteration in HCC and a hallmark of human cancer^6,7^.

TERT is the catalytic component of telomerase and extends telomeres with its RNA partner (telomerase RNA component). TERT can directly regulate the various hallmarks of cancer, which include sustaining proliferative signaling, evading growth suppressors, inducing angiogenesis, resisting cell death, activating invasion and metastasis, tumor-promoting inflammation, and enabling replicative immortality^8^. What’s more, TERT also acts as a regulator of the transcription of genes involved in cancer cell growth and proliferation, independently of its role in telomeres^9^. TERT is highly transcribed in germ cells, stem cells, and cancer cells, but is barely detectable in most somatic cells^10^. In cancer cells, TERT is transcriptionally reactivated by oncogenic transcription factors such as Myc, NF-κB, β-catenin, and SP1^8,11,12^, and TERT expression is elevated in HCC patients and positively correlated with the incidence and development of HCC^13,14^. However, the detailed molecular mechanism of TERT transcription activation in HCC is not very clear. A key question is whether specific oncogenic transcription factors or coactivators activate the transcription of TERT in HCC to promote HCC development.

Nuclear receptor coactivators-3 (NCOA3, also known as SRC-3 or AIBI) is a member of the steroid receptor coactivator (SRC)/p160 gene family, and it was identified from an amplified region on the long arm of chromosome 20 (20q) in breast cancer tissue^15^. NCOA3 protein has multiple structural and functional domains, including an N-terminal basic helix–loop–helix-Per/ARNT/Sim (bHLH-PAS) domain, a nuclear receptor-interacting domain, and a C-terminal transcriptional activation domain (AD) with histone acetyltransferase (HAT) and p300/CBP binding activities^16^. The SRC/p160 gene family has three homologous members, NCOA1/SRC-1, NCOA2/SRC-2, and NCOA3/SCR-3^17^. Unlike NCOA1 and NCOA2, NCOA3 mediates not only the transcriptional activity of nuclear receptors, but also other transcription factors, such as SP1, c-Myc, NF-κB, HIF1α, CREB, AP-1, E2F1, and PEA3^18–22^.

SP1, a key transcription factor of TERT, can bind to the GC-box of the TERT promoter and cooperate with c-Myc to active TERT transcription. Some regulators, including tumor activators and suppressors, can regulate SP1 binding activity to TERT promoter to enhance or inhibit TERT transcription in HCCs^23–26^.

NCOA3 is highly expressed in many human cancers, including breast, prostate, lung, pancreas, and colorectal carcinoma^27–29^. Overexpression of NCOA3 has been reported to enhance cell proliferation and invasion to promote HCC progression^30^, but the exact molecular mechanism of NCOA3-regulated HCC growth remains elusive.

In this study, we discovered and identified a new mechanism involved hTERT regulation and HCC growth. We designed a 5′ biotin-labeled TERT promoter (−387 to −137) probe to pull down the TERT promoter binding proteins by biotin–streptavidin-beads pull-down (BSBP) method and identified NCOA3 as a novel TERT promoter binding protein binds to the −234 to −144 region of the TERT promoter in HCC cells. We demonstrated that NCOA3 enhanced TERT expression in HCC cells, and NCOA3/TERT signaling promoted HCC cell growth in vitro and in vivo. We further revealed that NCOA3 interacted with SP1 to regulate TERT expression and HCC cell growth. Finally, our study showed that both NCOA3 and TERT were highly expressed in HCC patients and associated with poor clinical outcomes. Thus, our results uncovered a novel mechanism of HCC growth regulation by activating the NCOA3/SP1/hTERT axis, and indicating that the NCOA3-Sp1-TERT axis could be a potential therapeutic target for HCC.

## Materials and methods

### Cell lines and cell culture

HCC cells (Hep3B, HepG2, BEL-7402, and SNU-449) were obtained from the American Type Culture Collection (ATCC, Manassas, VA), human immortalized hepatic epithelial cell lines LO2 was obtained from Sun Yat-Sen University Cancer Center. Cells were cultured in Dulbecco’s Modified Eagle Medium (Invitrogen, Carlsbad, CA) or RPMI-1640 (Gibco BRL, Grand Island, NY) supplemented with 10% fetal bovine serum (Invitrogen), 100 µg/ml penicillin, and 100 U/ml streptomycins. All cells were maintained in a humidified atmosphere and 5% CO_2_ at 37 °C.

### Antibodies and reagents

Anti-NCOA3 antibody (#2126), anti-SP1 (D4C3) antibody (#9389), and anti-Acetylated-Lysine antibody (#9441) were purchased from Cell Signaling Technology (Danvers, MA). Anti-TERT antibody (#NB110-89471) was from Novus Biologicals (Littleton, CO). Anti-NCOA3 ChIP grade antibody (#ab2782) was from Abcam (Cambridge, MA). Anti-GAPDH antibody (#10494-1-AP), anti-histone H3 antibody (#17168-1-AP), horseradish peroxidase (HRP)-conjugated Goat Anti-Rabbit IgG antibody (#SA00001-2), Alexa Fluor 488 conjugated Goat Anti-Mouse IgG antibody (#SA00006-1), Alexa Fluor 594 conjugated Goat Anti-Rabbit IgG antibody (#SA00006-4) were from Proteintech (Wuhan, China). Real-time quantitative polymerase chain reaction (qPCR) reagents (#QPK-201) were from TOYOBO (Shanghai, China). A dual-luciferase detection kit (E1910) was obtained from Promega (Madison, WI).

### BSBP assay

The TERT promoter binding proteins were analyzed by streptavidin–agarose pulldown as described previously^31^. Briefly, the 5′ biotin-labeled double-stranded DNA probe corresponding to nucleotide −378 to −137 of the TERT promoter region was synthesized (Sigma-Aldrich, St. Louis, MO), and nuclear protein extracts of HCC cells were prepared. Totally, 1 mg nuclear protein extract, 10 µg DNA probe, and 100 µl streptavidin–agarose beads (Sigma-Aldrich) were mixed well and incubated at 4 °C overnight. The mixture was then washed to eliminate proteins bound at the DNA probe nonspecifically, centrifuged at 500 × *g* to precipitate the TERT promoter fragment/binding protein complex. The TERT promoter fragment binding proteins were separated by sodium dodecyl sulfate-polyacrylamide gel electrophoresis (SDS-PAGE) and visualized by silver staining (Beyotime, Haimen, China).

### Mass spectrometry (MS)

The HCC specific TERT promoter binding protein band in the PAGE gel was cut out and bleached with 30% ACN/100 mM NH_4_HCO_3_. After reduction and alkylation, the proteins in the band were digested with MS-grade trypsin solution (Promega, Madison, WI) and analyzed by ultrafleXtreme^TM^ matrix-assisted laser desorption ionization-time of flight mass spectrometry (MALDI-TOF)/TOF mass spectrometer (Bruker, Germany).

### Chromatin immunoprecipitation (ChIP) assay

ChIP assay was performed as described in Carey’s protocol^32^. Briefly, the cells were fixed with 1% formaldehyde, and the cross-linking was quenched by glycine (final concentration 137.5 mM). DNAs were sonicated on ice into 300–1000 bp fragments. One-third of each sample was used as the DNA input control, and the remaining two-thirds were subjected to immunoprecipitation with anti-NCOA3 antibody or nonimmune rabbit IgG (Cell Signaling Technology). PCR was performed to amplify a 250 bp TERT promoter segment. The PCR products were resolved in a 2% agarose gel and visualized by Gel-Red staining. ChIP-qPCR was performed using 9 primer pairs covering −1518 to +40 of TERT promoter (Supplementary Table 1). The relative enrichment of each fragment was normalized to the input.

### Electrophoretic mobility shift assay (EMSA)

The biotin-labeled DNA probes of TERT promoter regions −234 to −144 and −696 to −456 were synthesized. The EMSA assay was performed following the standard protocol of the Pierce Light Shift kit. Briefly, the probes, HCC cell nuclear extracts, and NCOA3 antibody were incubated at 25 °C for 20 min for a binding reaction. The NCOA3-probe complexes and free probes were separated in a 4% polyacrylamide gel and transferred to a nylon membrane. After ultraviolet cross-linking, the nylon membrane was treated with EMSA blocking buffer and then incubated with streptavidin–HRP conjugated solution. The bands were detected with ECL solution by Molecular Imager ChemiDoc^™^ XRS + and analyzed using the Image Lab software (Bio-Rad, Hercules, CA).

### Promoter reporters and dual-luciferase assay

To detect the regulation of NCOA3 on TERT promoter activity, truncation fragments of the TERT promoter (−902 to +40, −321 to +40, −234 to +40, −144 to +40, −70 to +40, −40 to +40) were amplified and inserted into SacI and HindІІІ sites of the firefly luciferase vector pGL4.10 (Promega, Madison, WI). and renilla luciferase reporter vector pRL-TK served as a control. The primers were shown in Supplementary Table 1. The HCC cells with NCOA3 overexpression or knockdown and the control cells were seeded into 96-well plates (2 × 10^4^ cells/well) and transfected with pGL4.10-TERT-truncation/pRL-TK (30:1–50:1) plasmids with Lipofectamine 3000 (Invitrogen, Carlsbad, CA). At 36 h after transfection, cells were lysed, and the dual-luciferase assay was performed according to the introduction of the Dual-Luciferase^®^ Reporter Assay System (Promega).

### Quantitative PCR (qPCR)

For RT-qPCR, total RNA was isolated using TRIZOL Reagent (Invitrogen, Carlsbad, CA), and cDNA was synthesized using Rever Tra Ace qPCR RT Kit (TOYOBO #FSQ-101, Shanghai, China) for qPCR. For ChIP-qPCR, the ChIP DNA fragments and input genomic DNAs served as temples. qPCR was performed with the SYBR Green PCR master mix (Applied Biosystems, Waltham, MA), and the amplification signals were detected by CFX96 Touch^TM^ (Bio-Rad, Hercules, CA) and analyzed by CFX Manager 3.0 (Bio-Rad). Target gene relative expression level was calculated by 2^−ΔCT^ (ΔCT = CT_Target gene_ − CT_GAPDH_) and normalized to the relative expression level detected in control cells. Each sample was tested in triplicate.

### Western blot

The HCC specimens and cells were homogenized with RIPA lysis buffer (Beyotime, Haimen, China) or Complete Lysis-M reagent (Roche, Indianapolis, IN) supplemented with cocktail protease inhibitors (Roche). The Protein concentration was detected by BCA assay (Pierce, Waltham, MA), and the proteins were separated by SDS-PAGE and transferred to polyvinylidene difluoride membranes. The membranes were blocked and sequentially incubated with primary antibodies and HRP-conjugated secondary antibody. The proteins were detected using an ECL solution.

### Lentivirus production and cell transfection

To establish HCC cells with stable NCOA3 overexpression, we constructed lentivirus pLV[Exp]-Puro-CMV-NCOA3-IRES:EGFP which encoded a full-length human NCOA3 gene, and pLV-CMV-EGFP-PGK-Puro as empty vector control. The lentivirus or the empty vector were transfected into 293FT cells together with lentivirus packing plasmids (pMDL:VSV-G:REV = 5:3:2) for NCOA3 lentivirus packing. The virus was then harvested to infect HCC cells, and stable clones with NCOA3 overexpression were selected after 2 weeks with 0.7–2 µg/ml puromycin. The expression of NCOA3 in the clones was determined by RT-qPCR and western blot. The hU6-NCOA3-EGFP-IRES-puromycin and negative control lentiviral vector containing non-silencing short hairpin RNA lentivirus plasmid was obtained from Genechem Company Ltd. (Shanghai, China). Two target sequences (5′-GCGCCAGAGATATGAAACA-3′ and 5′-GGCAGGGAGTTATTGATAA-3′) were used to knockdown NCOA3 expression. The efficiency of RNA interference was determined by RT-qPCR and Western Blot.

### Cell viability assay

Cells were seeded in 96-well plates (2 × 10^3^ cells/well). The viability of HCC cells was assessed by MTS assay (Promega, Madison, WI). The absorbance at 490 nm of each well was measured with a microplate reader. Each group included six replicates and three independent experiments were performed.

### Colony formation assay

Cells were seeded in 6-well plates (100–1000 cells/well) and cultured for 1–2 weeks. The colonies were fixed with 4% paraformaldehyde for 15 min at room temperature, stained with 2% crystal violet for 30 min, and counted under a microscope. Three independent experiments were performed.

### In vivo xenograft experiments

All animal procedures were performed following the Guide for the Care and Use of Laboratory Animals (NIH publication Nos. 80-23, revised 1996) and the Institutional Ethical Guidelines for Animal Experiments developed by Sun Yat-Sen University. Totally, 4–5-week old female SPF BALB/c nude mice were obtained from Beijing Vital River Laboratory Animal Technology Co., Ltd. The mice were subcutaneously injected with 3 × 10^6^ Hep3B cells (5 mice for each group). The length (*L*), width (*W*), and height (*H*) of the tumors were measured with calipers every 4 days, and the tumor volume (*V*) was calculated as *V* = *πLWH*/6. The standard streptavidin–biotin–peroxidase complex method was used for tumor xenograft immunohistochemical (IHC) staining. Briefly, after deparaffinization, blocking, and antigen retrieval, the tumor sections were incubated in a 1:100 dilution of NCOA3 or TERT-specific antibody at 4 °C overnight in a humidified chamber. After washing, tumor sections were incubated with HRP-conjugated anti-goat antibody (DakoCytomation, Carpentaria, CA) for 30 min at room temperature. Finally, 3,5-diaminobenzidine (DAB) substrate was used for color development, followed by Mayer’s hematoxylin counterstaining.

### IHC assay

HCC tissue microarray was purchased from Shanghai Outdo Biotech Co., Ltd. (OD-CT-DgLiv01-012), which contained 55 HCC tissues and 45/55 HCC patients and had follow-up outcomes. The other 12 HCC patients from Sun Yat-Sen University Cancer Center also were included to analyze clinical outcomes. Formalin-fixed tumor specimens were deparaffinized in xylene (5 min, twice), sequentially rehydrated in 100%, 95%, 80%, and 70% alcohols and distilled water. The slides were treated with 3% H_2_O_2_ for 15 min and antigen retrieval was performed in sodium citrate buffer (10 mM sodium citrate, 0.05% Tween 20, pH 6.0) at 121 °C for 5 min. The slides were then blocked with 5% bovine serum albumin (BSA) for 30 min and incubated with primary antibodies overnight. After three washings with phosphate-buffered saline (PBS), the slides were incubated with HRP-conjugated secondary antibody for 30 min, treated with DAB for color development, and counterstained with hematoxylin. For IHC scores, the percentage (0–100%) of stained tumor cells was multiplied by the intensity (0, 1, 2, or 3) to achieve a score between 0 and 300. All microscopy analyses were conducted by blinded experimenters.

### Co-immunoprecipitation (Co-IP) assay

To investigate the interaction between NCOA3 and transcription factors in HCC cells, Co-IP assay was performed. Totally, 5 × 10^6^ HCC cells were harvested, washed three times with cold PBS, and treated with Hypotonic Buffer (20 mM Tris-HCl, pH 7.4, 10 mM NaCl, 3 mM MgCl_2_·6H_2_O) for 15 min on ice. Totally, 10% NP40 was added and vortexed for 15 s. The cells were pelleted and treated with cell extraction buffer (100 mM Tris, pH 7.4, 2 mM Na_3_VO_4_, 100 mM NaCl, 1% Triton X-100, 1 mM EDTA, 10% glycerol, 1 mM EGTA, 0.1% SDS, 1 mM NaF, 0.5% deoxycholate, 20 mM Na_4_P_2_O_7_·10H_2_O) supplemented with cocktail protease inhibitors in ice for 30 min. After centrifugation, the supernatant was collected and pre-cleared with 50% protein A/G agarose beads (Millipore, Billerica, MA) for 1 h. Primary antibody was then added in for incubation overnight, and new protein A/G agarose beads were used to pull down proteins interacting with NCOA3.

### IF staining

Cells were fixed with 100% methanol (pre-chilled at −20 °C) at room temperature for 5 min and permeabilized with PBS containing 0.2% Triton X-100 for 10 min. After three 5-min washings with PBS, the cells were blocked with 1% BSA and 22.52 mg/ml glycine in PBST for 30 min and incubated with primary antibodies in PBST containing 1% BSA in a humidified chamber overnight at 4 °C. After three washings with PBS, fluorescence-conjugated secondary antibodies in PBST containing 1% BSA were added to the cells and incubated for 1 h at room temperature in the dark. NCOA3, TERT, and SP1 expression in HCC cells were observed under a confocal microscope.

### Statistical analysis

Data were presented as the mean ± standard deviation of at least three independent experiments. Statistical analysis was carried out using SPSS 11.0 software (SPSS Inc., Chicago, IL). Statistical tests were justified as appropriate. *P* < 0.05 was considered significant.

## Results

### NCOA3 was identified as a TERT promoter binding protein in HCC cells

**Fig. 1:**
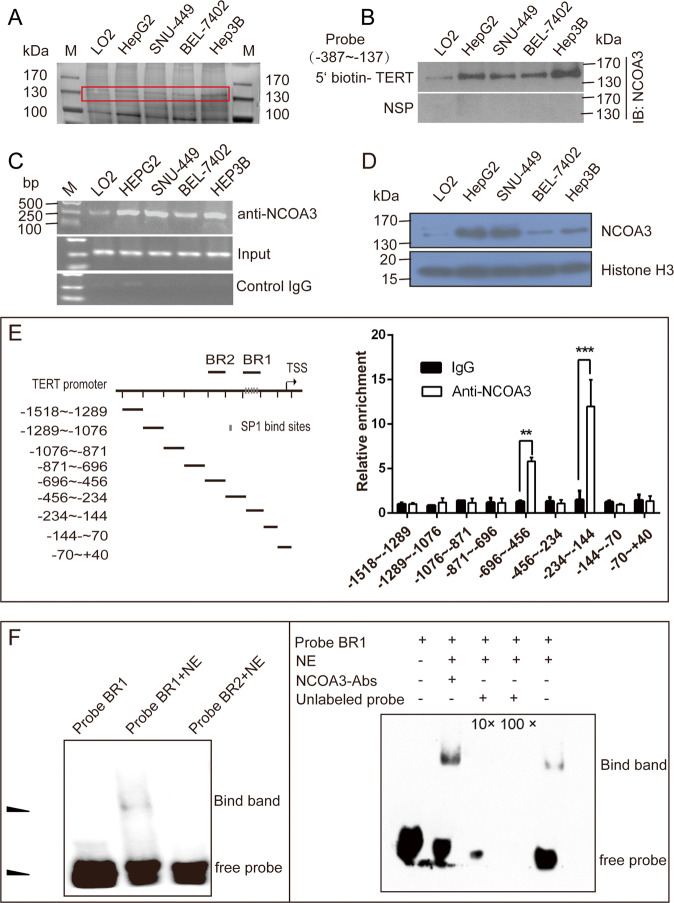
NCOA3 was identified as a new TERT promoter binding protein in HCC cells. **A** Potential TERT promoter binding proteins were pulled down using a 5′ biotin-labeled TERT promoter (−387 to −137) double-stranded DNA probe and streptavidin-beads in four HCC cell lines (HepG2, SNU-449, BEL-7402, and Hep3B) and one immortalized liver cell line (LO2). The proteins were separated by SDS-PAGE and visualized by silver staining. The box indicated the protein band that was enriched in HCC cells and processed for MS analysis. **B** NCOA3 was detected in the pull-down mixture by the 5′ biotin-labeled TERT promoter probe or a nonspecific probe (NSP) by western blot. **C** ChIP assay was performed in LO2 and HCC cells with NCOA3 antibody and TERT promoter-specific primers. The PCR products were separated in a 2% agarose gel, and IgG was used as a negative control. **D** Protein level of NCOA3 in the nuclear extracts of LO2 and HCC cells. Histone H3 served as a control. **E** Illustration of the primer pairs for the ChIP-qPCR assay. BR1 binding region 1, BR2 binding region 2, TSS transcription start site. Enrichment of NCOA3 at different regions of TERT promoter was detected by ChIP-qPCR in Hep3B cells. Data were shown as means ± SD. **P < 0.01. ***P < 0.001, Student’s *t* test. Each sample was tested in triplicate. **F** EMSA assay was performed with 5′ biotin-labeled TERT promoter probe BR1 (−234 to −144) or BR2 (−696 to −456) and the nuclear extract of Hep3B cells. The upper arrowhead denoted the probes bound by NCOA3, and the lower arrowhead denoted free probes. NE nuclear extract. The supper shift assay was performed using NCOA3 specific antibody, BR1, and the nuclear extract of Hep3B cells. Unlabeled TERT promoter probes were added as the competitor.

The transcription of TERT was over-activated in HCC cells. In order to discover and identify the specific transcriptional factors that regulate TERT expression and cell growth in HCC, we used the BSBP method coupled with MALDI–TOF/TOF MS approach to identify the TERT promoter binding proteins in HCC cell lines (HepG2, Hep3B, BEL-7402, SNU-449) and immortalized liver cell line (LO2). The non-liver cancer cell line (HEK293) was used as a negative control. We found that NCOA3, a potential candidate, differentially bound to the TERT promoter in HCC cells (Fig. 1A, Fig. S1A). To validate that NCOA3 bound to the TERT promoter, we detected NCOA3 with its specific antibody in the nuclear proteins pulled down by the TERT promoter probe in LO2 immortalized liver cells and HCC cells. As is shown in Fig. 1B, a higher level of NCOA3 was pulled down in the HepG2, SNU-449, BEL-7402, and Hep3B HCC cells than in LO2 cells. Moreover, a ChIP assay was performed to confirm that NCOA3 bound to TERT promoter in LO2 and HCC cells, and the results showed that NCOA3 had much stronger binding at the endogenous TERT promoter in HCC cells compared to LO2 cells (Fig. 1C). We detected the mRNA expression of TERT in HCC cells and LO2 cells and found that TERT expression was higher in HCC cells than in LO2 cells (Fig. S1B). Consistently, the expression of NCOA3 was higher in HCC cells (Fig. 1D).

Next, to identify the exact binding locus of NCOA3 at the TERT promoter, we performed ChIP-qPCR with nine pairs of primers that covered the 1500 bp region upstream of the TERT transcription start site (TSS). Our results showed that NCOA3 was enriched at the −234 to −144 and −696 to −456 regions of TERT promoter in Hep3B cells (Fig. 1E), and EMSA assay confirmed that NCOA3 bound to the −234 to −144 region of TERT promoter (Fig. 1F). Accordingly, these data validated NCOA3 as a new TERT promoter binding protein in HCC cells.

### NCOA3 transcriptionally activated TERT expression in HCC cells

**Fig. 2:**
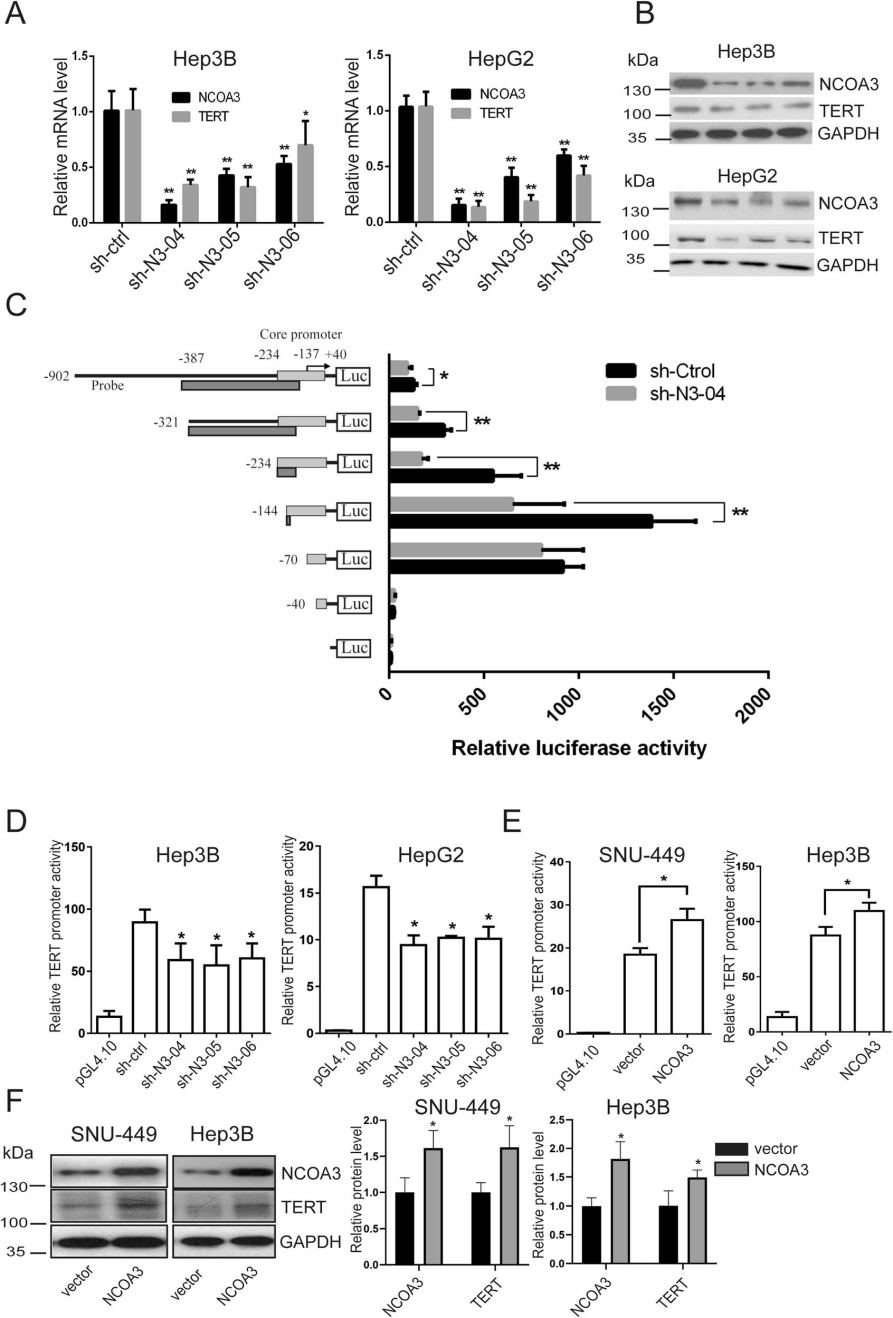
NCOA3 activated TERT promoter and increased TERT expression in HCC cells. **A**, **B** mRNA (**A**) and protein (**B**) levels of NCOA3 and TERT in Hep3B and HepG2 cells with NCOA3 knockdown. **C** Activity of different fragments of TERT promoter was determined by dual-luciferase reporter assay with transfection of pGL4.10-TERT-truncation/pRL-TK plasmids as the ratio of 50:1 in Hep3B cells without and with NCOA3 knockdown. The firefly luciferase activity was normalized to the renilla luciferase activity. **D** TERT promoter activity was decreased with NCOA3 knockdown. The TERT promoter activity was detected using the −234 ~ +40 fragment of TERT promoter according to (**C**) by transfection of pGL4.10-TERT-truncation/pRL-TK plasmids as the ratio of 30:1 in NCOA3 knockdown Hep3B and HepG2 cells. **E** TERT promoter (−234 to +40) activity was increased in SNU-449 and Hep3B cells with NCOA3 overexpression. **F** Protein level of NCOA3 and TERT in SNU-449 and Hep3B cells with NCOA3 overexpression. All of the data in the figure were from at least three independent experiments and shown as means ± SD. **P < 0.01. ***P < 0.001, Student’s *t* test.

To test whether NCOA3 bound to the TERT promoter to activate TERT transcription, we established HCC cell lines with stable knockdown or overexpression of NCOA3 by lentivirus infection. We found that knockdown of NCOA3 decreased TERT mRNA and protein levels in HepG2 and Hep3B cells (Fig. 2A, B), while overexpression of NCOA3 enhanced TERT expression in SNU-449 and Hep3B cells (Fig. 2F). To examine whether NCOA3 regulated TERT promoter activity, we constructed a series of luciferase reporter plasmids that contained different segments of the −902 to +40 region of TERT promoter. Our results indicated that the −234 to −40 region was essential for potent TERT promoter activity (Fig. 2C, sh-Ctrol). Also, knockdown of NCOA3 dramatically decreased the promoter activity of the −234 to +40 and −144 to +40 fragments, but not the activity of the −70 to +40 fragment (Fig. 2C), which suggested that the −234 to −70 region of TERT promoter contained a major NCOA3-binding site. This was in accordance with our ChIP-qPCR and EMSA results (Fig. 1E, F). We further confirmed the role of NCOA3 in regulating the activity of TERT promoter in HCC cells with the luciferase reporter plasmid that had the −234 to +40 fragment. Knockdown of NCOA3 significantly decreased the TERT promoter activity in Hep3B and HepG2 cells (Fig. 2D). In contrast, overexpression of NCOA3 remarkably enhanced the TERT promoter activity in SNU-449 and Hep3B cells (Fig. 2E, F). Taken together, these data indicated that NCOA3 bound to the TERT promoter and activated TERT transcription.

### NCOA3 promoted tumor cell growth via TERT signaling in HCC cells and mouse HCC xenograft model

**Fig. 3:**
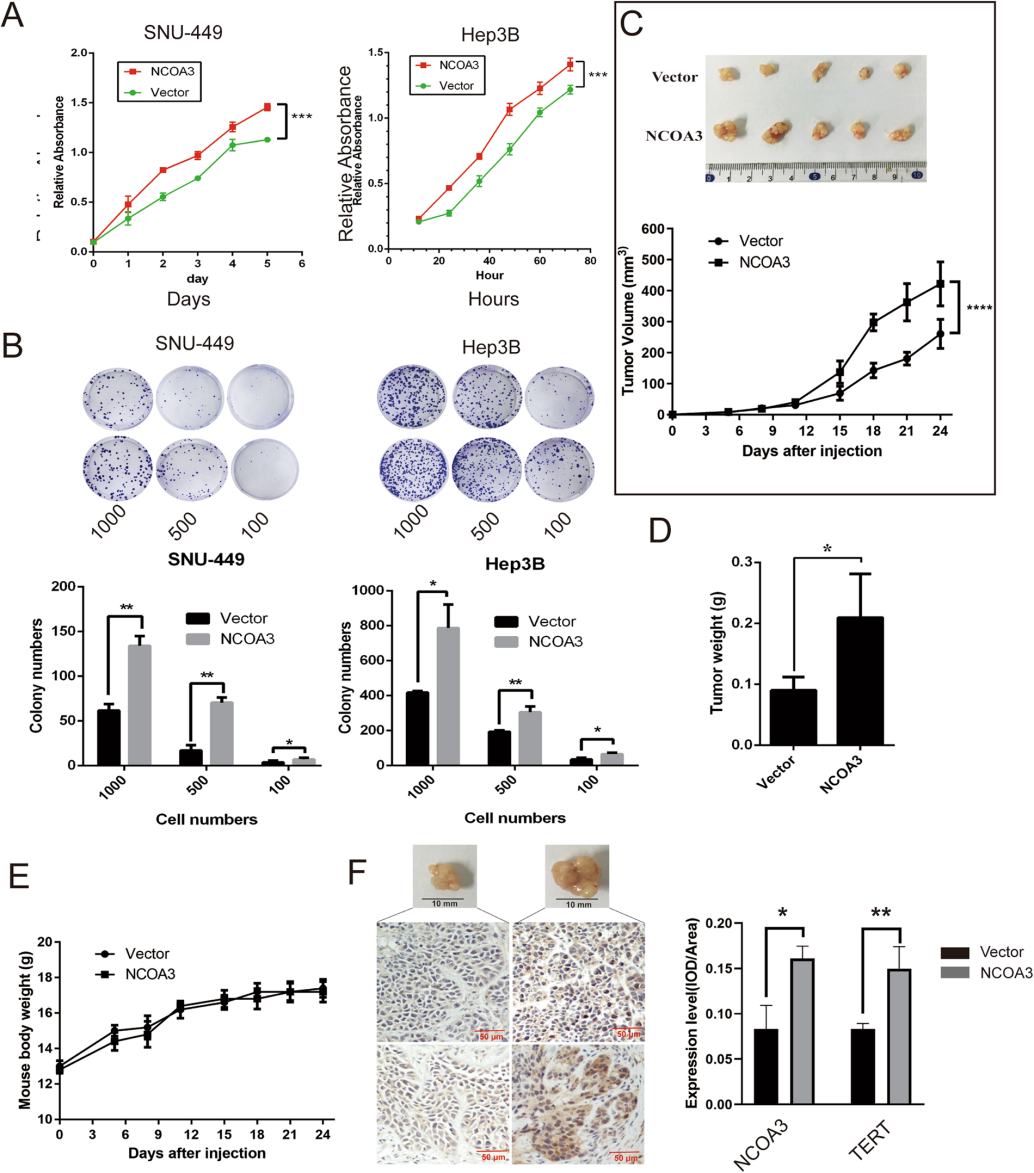
Overexpression of NCOA3 promoted HCC cell growth in vitro and in vivo. **A** Viability of SNU-449 and Hep3B cells with NCOA3 overexpression was determined by MTS assay. **B** The colony formation ability of SNU-449 and Hep3B cells with NCOA3 overexpression. The colonies were stained by crystal violet and counted. **C** Nude mice were subcutaneously injected with Hep3B cells with vector or NCOA3 overexpression. Images of the HCC tumor xenograft from each mouse (*n* = 5 mice/group) were captured, tumor volumes were recorded every 3 days, **D** tumor weights were analyzed, **E** mouse body weights were monitored, and **F** the expression of NCOA3 and TERT in tumor tissues were detected by IHC staining. The data were shown as means ± SD, * *P* < 0.05, **P < 0.01. ***P < 0.001, Student’s *t* test.

To investigate the function of NCOA3 in HCC cell growth, we first established SNU-449 and Hep3B cells with stable NCOA3 overexpression. As shown in Fig. 3A, B, overexpression of NCOA3 effectively enhanced HCC cell viability and colony formation. Moreover, overexpression of NCOA3 increased the size and weight of tumors in a mouse HCC xenograft model (Fig. 3C, D), though the bodyweight of the mice remained unchanged (Fig. 3E). IHC staining showed that the expression of TERT was elevated in xenograft tumors with NCOA3 overexpression (Fig. 3F).

**Fig. 4:**
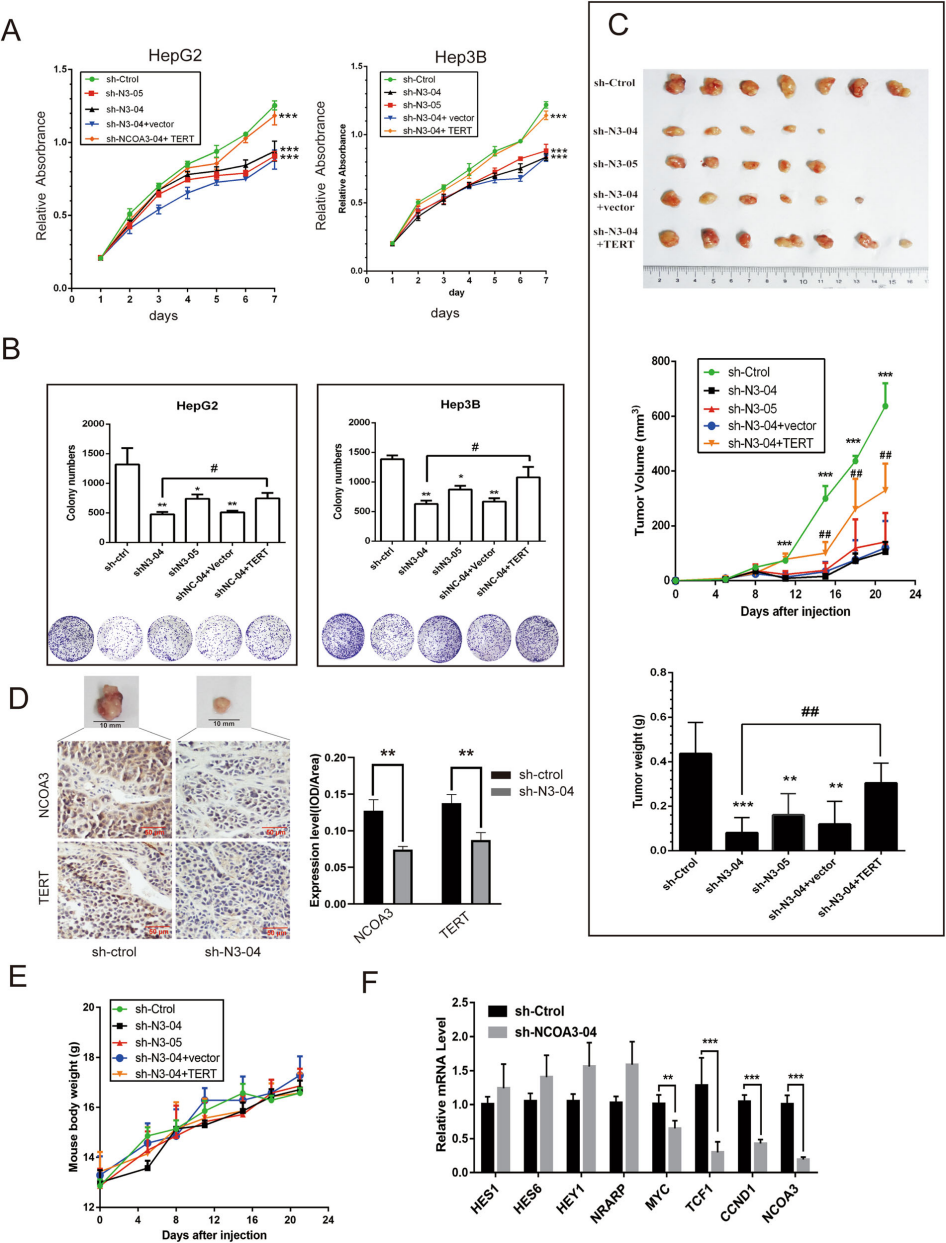
Knockdown of NCOA3 inhibited HCC cell growth in vitro and in vivo. **A** Viability of HepG2 and Hep3B cells with NCOA3 knockdown. **B** The colony formation ability of HepG2 and Hep3B cells with NCOA3 knockdown. **C** Nude mice were subcutaneously injected with Hep3B cells with nonspecific shRNA (sh-Ctrol), NCOA3-specific shRNAs (sh-N3-04, sh-N3-05), sh-N3-04 + vector, or sh-N3-04 + TERT overexpressing plasmid (TERT). Images of the HCC tumor xenograft from each mouse (n = 5 mice/group) were captured, tumor volumes were recorded every 4 days, tumor weights were analyzed, **D** the expression of NCOA3 and TERT in tumor tissues were detected by IHC staining, and mouse body weights were monitored (**E**). **F** mRNA levels of the Notch target genes (HES1, HES6, HEY1, and NRARP), Wnt target genes (MYC, TCF-1, and CCND1), and NCOA3 in Hep3B cells with NCOA3 knockdown were measured by RT-qPCR. The data were shown as means ± SD, *P < 0.05, **P < 0.01. ***P < 0.001, ^#^*P* < 0.05, ^##^*P* < 0.01, Student’s *t* test.

Next, we established HepG2 and Hep3B cells with stable knockdown of NCOA3 and found that NCOA3 knockdown significantly inhibited HCC cell viability and colony formation (Fig. 4A, B). Interestingly, overexpression of TERT partly rescued the inhibition of cell viability and colony formation caused by NCOA3 knockdown (Fig. 4A, B), which suggested that NCOA3 promoted HCC cell growth via TERT signaling. Consistently, results from our HCC xenograft model showed that NCOA3 knockdown decreased the size and weight of tumors without affecting the mouse body weight, and the decrease was partly reversed by TERT overexpression (Fig. 4C, E). Also, IHC staining showed that the expression of TERT was decreased in xenograft tumors when NCOA3 was knocked down (Fig. 4D).

It is known that TERT activates Wnt signaling independently of its telomerase reverse transcriptase activity^27^. To further examine whether NCOA3 promoted HCC growth via the TERT signaling pathway, we examined the expression of the Wnt target genes in NCOA3 knockdown Hep3B cells and found that knockdown of NCOA3 decreased the expression of the Wnt targets MYC, TCF-1, and CCND1, but had no effect on the expression of the Notch target genes HES1, HES6, HEY1, and NRARP (Fig. 4F). Collectively, these in vitro and in vivo results demonstrated that NCOA3 promoted HCC cell growth via TERT signaling.

### NCOA3 cooperated with SP1 to promote TERT expression and HCC cell growth

**Fig. 5:**
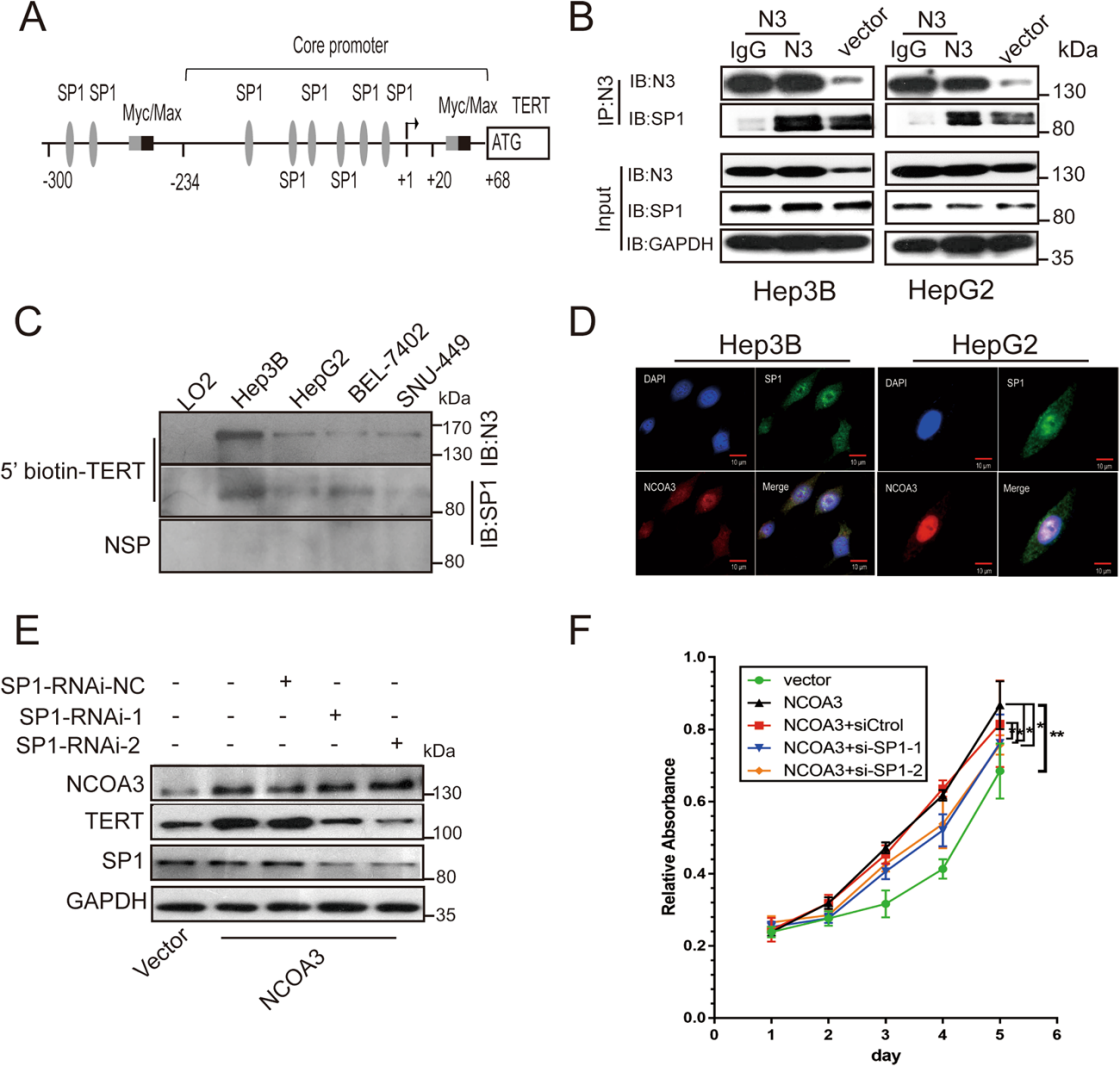
NCOA3 interacted with SP1 to promote TERT expression and HCC cell growth. **A** Illustration of the SP1 binding sites in the TERT promoter. **B** The interaction between NCOA3 and SP1 was tested by co-IP in Hep3B and HepG2 cells without (vector) or with NCOA3 overexpression (N3). NCOA3 antibody was added to the nuclear extracts to precipitate proteins interacting with NCOA3, while IgG was used as a negative control. SP1 was then detected by its specific antibody in the precipitates. **C** The 5′ biotin-labeled TERT promoter probe was pulled down by NCOA3 antibody in immortalized LO2 and HCC (Hep3B, HepG2, BEL-7402, and SNU-449) cells, while a nonspecific probe (NSP) was used as a negative control. SP1 was detected by its specific antibody in the precipitates. **D** Immunofluorescence of NCOA3 (red) and SP1 (green) in Hep3B and HepG2 cells. **E** TERT expression was detected in Hep3B cells without (Sp1-RNAi-NC) or with SP1 knockdown (Sp1-RNAi-1, Sp1-RNAi-2). **F** Viability of Hep3B cells with NCOA3 overexpression and SP1 knockdown. The data were shown as means ± SD, * *P* < 0.05, **P < 0.01, Student’s *t* test from three independent experiments.

As a transcriptional coactivator, NCOA3 interacts with many bHLH (basic helix–loop–helix) transcription factors like HIF1α through its N-terminal domain to activate target gene expression^22,28^. In order to identify the exact transcription factor(s) that interacted with NCOA3 to activate TERT transcription, we analyzed the transcription factors binding to the −300 ~ +68 region of TERT promoter and found that this region had many SP1-binding sites (Fig. 5A). Considering that SP1 is a bHLH domain-containing transcription factor, and it is critical for TERT transcription activation, we hypothesized that NCOA3 interacted with SP1 to activate TERT transcription and promote HCC cell growth. Endogenous Co-IP experiment revealed that NCOA3 interacted with SP1 in Hep3B and HepG2 cells, and this interaction was enhanced by NCOA3 overexpression (Fig. 5B). Also, SP1 was detected on the 5′ biotin-labeled TERT promoter probe (Fig. 1B) pulled down by NCOA3 antibody in HCC cells (Fig. 5C). In addition, immunofluorescence (IF) showed that NCOA3 was co-localized with SP1 in the nuclei of Hep3B and HepG2 cells (Fig. 5D). We further explored whether NCOA3 and SP1 co-regulated TERT expression and found that knockdown of SP1 in Hep3B cells abolished the TERT expression induced by NCOA3 overexpression (Fig. 5E). Consistently, knockdown of SP1 partially inhibited the growth of Hep3B cells caused by NCOA3 overexpression (Fig. 5F). Taken together, these results indicated that NCOA3 interacted with SP1 to promote TERT expression and HCC cell growth.

### The expression of NCOA3 and TERT were elevated in HCC tissues

**Fig. 6:**
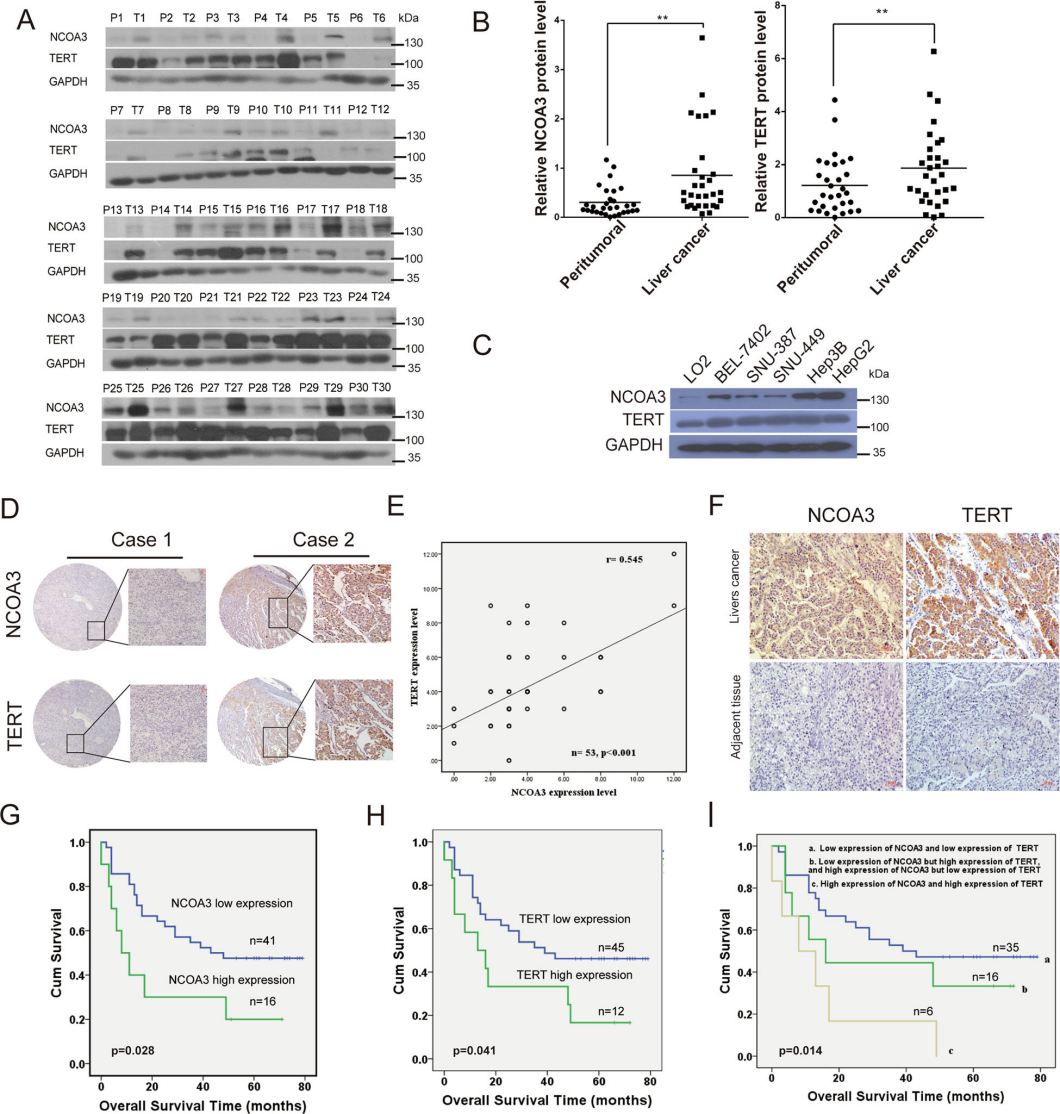
The expression of NCOA3 and TERT in HCC tissues and were associated with poor clinical outcomes in HCC patients. **A** Expression of NCOA3 and TERT in the peritumor (P) and tumor (T) tissues was detected by western blot in 30 human HCC tissues. **B** Quantification of NCOA3 and TERT protein expression in (**A**), GAPDH as an internal control, data were shown as means ± SD, **p < 0.01, *n* = 30, Student’s *t* test. **C** Protein level of NCOA3 and TERT in immortalized LO2 and HCC (BEL-7402, SNU-387, SNU-449, Hep3B, and HepG) cells. **D** Representative images of IHC staining of NCOA3 and TERT in HCC tissues using a tissue microarray. **E** The expression of NCOA3 and TERT were positively correlated in HCC tissue microarray. The statistical test indicated a significant (*p* < 0.001) positive correlation, by a two-tailed Pearson correlation test, *n* = 53, *r* = 0.545. **F** Representative images of IHC staining of NCOA3 and TERT in HCC and adjacent normal tissues. **G**–**I** Prognosis of HCC patients with high or low expression of NCOA3 (**G**), TERT (**H**), and NCOA3 + TERT (**I**) was shown by Kaplan–Meier overall survival curves. Fifty-seven HCC tissues including 45 from the tissue microarray (Fig. 6E, 45/53) and 12 from HCC patients in Fig. 6A, (12/30) were used to analyze clinical outcomes in Fig. 6G–I. NOCA3 low expression (*n* = 41), blue line, NOCA3 high expression (*n* = 16), green line, *p* = 0.028 in Fig. 6G; TERT low expression (*n* = 45) blue line, NOCA3 high expression (*n* = 12), green line, *p* = 0.041 in Fig. 6H; NOCA3/TERT low expression (*n* = 35), blue line, NOCA3/TERT high expression (*n* = 6), copper-colored line, NOCA3 low/TERT high expression and NOCA3 high/TERT low expression (*n* = 16), green line, *p* = 0.014; Kaplan–Meier test.

Analysis of the TCGA database for TERT gene variations in liver cancer patients revealed that TERT gene amplification occurred in about 4% (23/587) of the liver cancer patients (Fig. S2A). However, TERT mRNA was upregulated in over half (338/587) of the patients (Fig. S2B), suggesting that the increased expression of TERT in HCC was caused by elevated TERT transcription. We then detected the NCOA3 and TERT protein expression in the tumor and peritumoral tissues of 30 HCC patients from the Sun Yat-Sen University Cancer Center. Our results showed that both NCOA3 and TERT had higher expression in the tumor tissues (Fig. 6A, B), and the expression of NCOA3 and TERT were, respectively, elevated in 88% (26/30) and 78% (23/30) of the HCC tissues. Moreover, the expression of TERT was positively correlated with the expression of NCOA3 in HCC patients (*r* = 0.391, *p* = 0.002) (Fig. S2C). Consistently, NCOA3 and TERT had higher expression in HCC cells (BEL-7402, SNU-387, SNU-449, Hep3B, and HepG2) compared with the LO2 immortalized liver cells (Fig. 6C). We also analyzed the correlation between NACO3, SP1 and TERT mRNA expression in liver cancer patients in the GEO database (GEO: GSE10143), the mRNA level of SP1 was a positive correlation with the NCOA3 (*R*^2^ = 0.7367, *p* < 0.0001) and TERT (*R*^2^ = 0.0.401, *p* < 0.0001) (Fig. S2D). Moreover, the Kaplan–Meier survival analysis of GEO data showed that the patients with high mRNA expression of SP1, NACO3, and TERT had significantly shorter survival times (Fig. S2E, F). These results indicated that the expression of SP1, NACO3, and TERT had a positive correlation in tumor tissues. Moreover, the combination of the expression of SP1, NACO3, and TERT may serve as a survival predictor in hepatocellular carcinoma.

### High expression of NCOA3, SP1, and TERT was associated with poor clinical outcomes in HCC patients

We further analyzed the clinical significance of NCOA3/TERT high expression in HCC patients. Tissue microarray data showed that the expression of NCOA3 and TERT were positively correlated in HCC patients (*n* = 53) (Fig. 6D, E), and were higher in tumor tissues than in adjacent nontumor tissues (Fig. 6F). Moreover, we analyzed the follow-up outcome data from the 45 patients in the tissue microarray and 12 other HCC patients (Fig. 6A, B), and found that the HCC patients (*n* = 57) with high expression of NCOA3(16/57) or TERT (12/57) had poor prognosis (Fig. 6G, H), and the patients with high expression of both NCOA3 and TERT(6/57) had the worst outcomes, whereas the patients with low expression of both NCOA3 and TERT (35/57) had the best outcomes (Fig. 6I). Furthermore, we also analyzed the prognosis of the 156 liver cancer patients with high and low expression of NCOA3/TERT/SP1 in the clinic date sets of the GEO database (GSE10143) and found the patients with high expression with NCOA3, SP1, or TERT have a poor prognosis comparison the HCC patients with low expression of this gene (Fig. S2E). And, the HCC patients with both high expression of SP1/NCOA3 and SP1/TERT have the worst prognosis compared with those with low SP1, NCOA3, and TERT expression (Fig. S2F).

Taken together, these results suggested that high expression of NCOA3/TERT/SP1 was a key factor to predict the prognosis of HCC patients.

## Discussion

Our study has for the first time identified NCOA3 as a transcriptional coactivator that bound to the −234 to −144 region of TERT promoter in HCC cells, regulated TERT expression through interaction with SP1, and thus promoted HCC cell growth via TERT signaling. We have also found that NCOA3/TERT high expression in HCC tissues is a poor prognosis factor for HCC patients. Our findings have revealed a novel role of NCOA3 in the regulation of TERT expression and HCC tumorigenesis and suggested that it could be a target for HCC therapy.

TERT is the catalytic subunit of telomerase that maintains the length of telomeres and thus plays a decisive role in cell immortalization^33,34^. In recent years, TERT has been reported to mediate many molecular events independently on its reverse transcriptase activity, which include activation of the Wnt/β-catenin signaling, induction of angiogenesis, regulation of DNA damage response, and inhibition of apoptosis in cancer development^35–40^. Therefore, TERT is regarded as a central regulator of the hallmarks of cancer, and therapeutics targeting both its canonical and noncanonical functions are suggested to be more effective in cancer treatment^9^.

Our analysis of TERT gene variations in liver cancer patients using the TCGA database revealed that amplification of the TERT gene occurred in only ~4% (23/587) of the liver cancer patients (Fig. S2A), which was consistent with the studies by other groups^30,41^. In contrast, the mRNA level of TERT was upregulated in over 55% (338/587) of the liver cancer patients (Fig. S2B), suggesting that the expression of TERT was mainly regulated on the transcription level. The TERT gene consists of 15 introns and 16 exons, and the TERT core promoter is located in −234 upstream of its TSS to +37 of the second exon^42^, containing enriched CpG dinucleotide sites, SP1 binding sites, and E-box, but lacks TATA box and CAAT box^43^. The transcription of TERT is regulated by many positive and negative factors, and SP1 and Myc are two common transcription factors that activate TERT expression in cancer cells^44,45^. SP1 is a zinc finger transcription factor that binds to the GC-rich region of TERT promoter^46^, while Myc belongs to the bHLH-zip (basic helix–loop–helix-zipper) protein family and forms a heterodimer with Max to bind at the E-box of TERT promoter^47^. Studies have suggested that the elevated TERT expression mediated by Myc/Max depends on SP1^45^. In this study, we discovered that NCOA3 bound to the −234 to −144 region of TERT promoter and activated TERT transcription in HCC cells (Figs. 1 and 2). Our further investigation showed that NCOA3 interacted with the transcription factor SP1 as a coactivator to promote TERT expression and HCC growth (Fig. 5).

NCOA3 is a nuclear receptor coactivator, and it interacts with other transcription factors via its N-terminal bHLH-PAS (basic helix–loop–helix–PER–ARNT–SIM) domain^48^. The bHLH-PAS family proteins are important in several protumor and antitumor pathways^49^. In this study, we found that NCOA3 had higher expression in HCC cells than in the LO2 immortalized hepatocytes (Fig. 6C), and in HCC tumor tissues than in peritumoral tissues (Fig. 6A, B). Moreover, our results showed that high expression of NCOA3 promoted TERT expression and HCC cell growth (Fig. 2 and Fig. 3). Considering that SP1 is a general transcription factor and interacts with bHLH proteins, we propose that the elevated expression of NCOA3 and its interaction with SP1 is crucial to the transcription activation of TERT in HCC. In addition, NCOA3 can recruit the acetyltransferase p300/CBP through its AD at the C-terminus^48^, so we speculate that NCOA3 may also activate TERT transcription by recruiting p300/CBP to make chromatin DNA more accessible to the RNA polymerase II transcription machinery.

Spatial and temporal modification and transporting of essential regulators between different cell compartments is one of the key events in cellular signal transduction, gene activation, and protein dynamics during various biological processes. The nuclear localization of NCOA3 depends on the phosphorylation and two nuclear localization signals in its N-terminal bHLH domain^50^. The stability of NCOA3 can affect the nuclear localization of NCOA3 through the proteasome-dependent pathway by recognizing two sites (K17 and R18) of the N-terminal bHLH domain of NCOA3^51^. We found that the amount of nuclear NCOA3 binding to the TERT promoter in different four HCC cell lines is not consistent with the total protein levels in these cells (Fig. 1D). The possible reason is that the phosphorylation modification and stability of NCOA3 do not constant in these cells, thereby leading to a different binding amount in the TERT promoter.

Somatic mutation in TERT promoter frequently occurs in HCC patients (59%), the most two hot mutation spots were −124 and −146 bp from the ATG start site^52^. We found that NCOA3 bound to the −234 to −144 bp from the transcription site of the TERT promoter (−291 to −201 bp from the ATG start site). And, the hot spots −124 resides the sequence of SP1 binding. So, the mutation in the TERT promoter may have a function that facilitates the recruitment of transcription factors or co-factors into the TERT promoter to activate its expression. However, the potential mechanism and clinical significance of TERT promoter mutation which enhances NCOA3 binding and active TERT expression and cell growth in HCC cells need further exploration.

## Supplementary information

Supplementary Table 1

Supplementary Figure S1

Supplementary Figure S2

Supplementary Figure Legends
