# Supplementary figures and images for: Targeting the NCOA3-SP1-TERT axis for tumor growth in hepatocellular carcinoma
Source: Cell Death Dis. 2020 Nov 25;11(11):1011. doi: 10.1038/s41419-020-03218-x (PMC7689448; doi:10.1038/s41419-020-03218-x)

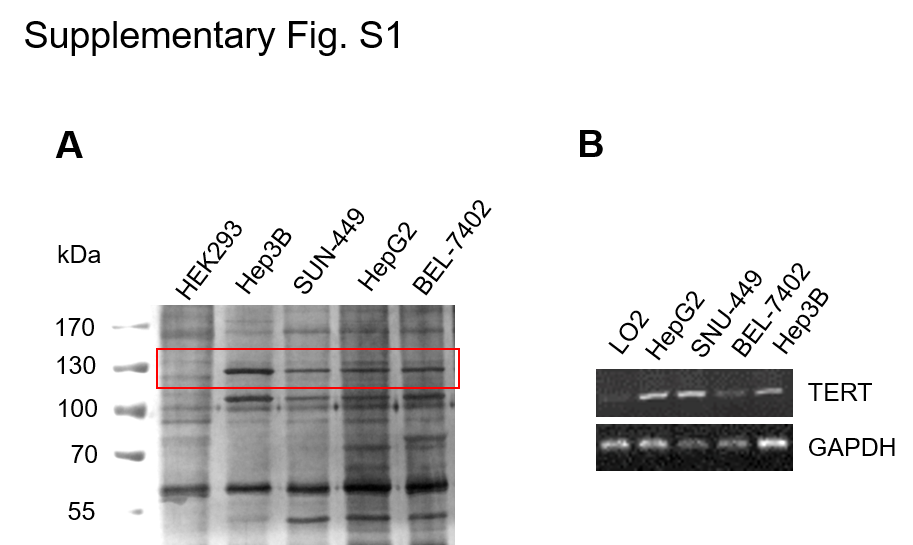

Supplement: Supplementary file 2 — Supplementary Figure S1 [file 41419_2020_3218_MOESM2_ESM.tif]

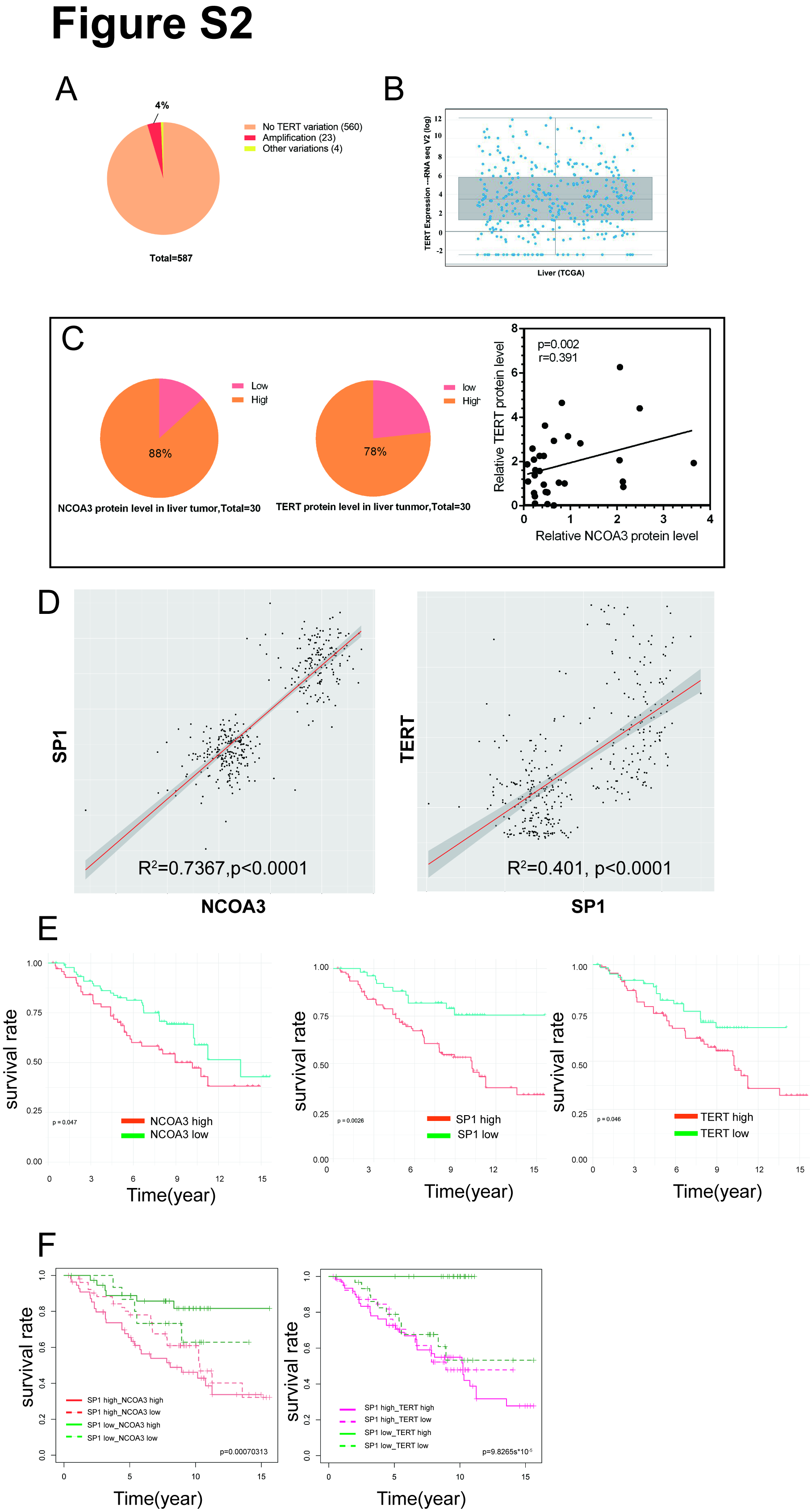

Supplement: Supplementary file 3 — Supplementary Figure S2 [file 41419_2020_3218_MOESM3_ESM.tif]
