# Supplementary material for: Targeting the NCOA3-SP1-TERT axis for tumor growth in hepatocellular carcinoma
Source: Cell Death Dis. 2020 Nov 25;11(11):1011. doi: 10.1038/s41419-020-03218-x (PMC7689448; doi:10.1038/s41419-020-03218-x)
Supplement: Supplementary file 4 — Supplementary Figure Legends [file 41419_2020_3218_MOESM4_ESM.doc]

**Supplementary Figure Legends**

**Supplementary Figure S1**. **NCOA3 differentially bound to TERT promoter in HCC cells.** (A) Potential TERT promoter binding proteins were pulled down using a 5’ biotin-labeled TERT promoter probe as in Figure 1A and streptavidin-beads in four HCC cell lines and non-liver cancer cell line, HEK293. The proteins were separated by SDS-PAGE and visualized by sliver staining. The box indicated the protein band that was enriched in HCC cells in Figure 1A. (B) TERT mRNA expression level was detected before the ChIP assay in Figure 1C in four HCC cell lines (HepG2, SNU-449, BEL-7402 and Hep3B) and one immortalized liver cell line (LO2), GAPDH expression as an internal control.

**Supplementary Figure S2. NCOA3/TERT/SP1 were highly expressed in HCC patients and predicted a poor prognosis in HCC patients**. **(A)** The ratio of TERT gene amplification in liver cancer patients (n=587) in TCGA database ([http://cancergenome.nih.gov/). **(B**](http://cancergenome.nih.gov/). (B)) TERT expression level in 587 liver cancer patients by RNA sequencing data for the TCGA database. **(C)** The percentage of HCC patient with NCOA3 high expression (left panel), TERT high expression (middle panel) and the correlation between with the NCOA3 and TERT expression in 30 HCC patients in Figure 6A, B by two-tailed Pearson correlation test, n=30, r=0.391, p=0.002. **(D)** The mRNA expression correlation of NCOA3/SP1 and TERT/SP1 in 156 liver cancer patients form the GEO database (GEO: GSE10143). The processed data form GSE10143 dataset was download form GEO database, the mRNA expression level was normalized and the data was visualized by ggplot2 in R environment. **(E, F)** The survival rate of the liver patients with NCOA3/SP1/TERT high or low expression. The clinic dataset form GEO database (GEO: GSE10143) was acquired using GEOquery package in R. The cutoff value of NCOA3, TERT and SP1 was calculated through OptimalCutpoints according to the patient’s survival. The gene high expression in the patients was defined as > cutoff value, then the Kaplan-Meier survival curve was painted using survival package in R.
